# Genome landscapes of rectal cancer before and after preoperative chemoradiotherapy

**DOI:** 10.7150/thno.37794

**Published:** 2019-09-21

**Authors:** Jie Yang, Yuan Lin, Ying Huang, Jing Jin, Shuangmei Zou, Xiaolong Zhang, Hongmin Li, Ting Feng, Jinna Chen, Zhixiang Zuo, Jian Zheng, Yexiong Li, Ge Gao, Chen Wu, Wen Tan, Dongxin Lin

**Affiliations:** 1 State Key Laboratory of Molecular Oncology, Department of Etiology and Carcinogenesis, National Cancer Center/National Clinical Research Center/Cancer Hospital, Chinese Academy of Medical Sciences and Peking Union Medical College, Beijing, China; 2 Beijing Advanced Innovation Center for Genomics (ICG), Biomedical Pioneering Innovation Center (BIOPIC), Peking University, Beijing, China; 3 State Key Laboratory of Protein and Plant Gene Research, School of Life Sciences, Center for Bioinformatics, Peking University, Beijing, China; 4 Department of Radiation Oncology, National Cancer Center/National Clinical Research Center/Cancer Hospital, Chinese Academy of Medical Sciences and Peking Union Medical College, Beijing, China; 5 Department of Pathology, National Cancer Center/National Clinical Research Center/Cancer Hospital, Chinese Academy of Medical Sciences and Peking Union Medical College, Beijing, China; 6 State Key Laboratory of Oncology in South China, Collaborative Innovation Center for Cancer Medicine, Sun Yat-sen University Cancer Center, Guangzhou, China

**Keywords:** whole-exome sequencing, mutation, copy-number variation, survival, rectal cancer

## Abstract

Resistance to preoperative chemoradiotherapy (CRT) is a major obstacle to cancer treatment in patients with locally advanced rectal cancer. This study was to explore genome alterations in rectal cancer under CRT stress.

**Methods**: Whole-exome sequencing (WES) was performed on 28 paired tumors collected before and after CRT from the same patients who did not respond to CRT treatment. Somatic point mutations and copy number variations were detected by VarScan2 and Exome CNVs respectively using paired tumor and blood samples. Somatic alterations associated with CRT resistance were inferred considering differences in significantly mutated genes, mutation counts and cancer cell fraction between matched pre- and post-CRT tumors. We employed SignatureAnalyzer to infer mutation signatures and PyClone to decipher clonal evolution and examine intratumoral heterogeneity in tumors before and after CRT. The associations between intratumoral heterogeneity and patients' survival were analyzed using the log-rank test and the Cox regression model.

**Results**: (i) Recurrent mutations in *CTDSP2*, *APC*, *KRAS*, *TP53* and *NFKBIZ* confer selective advantages on cancer cells and made them resistant to CRT treatment. (ii) CRT alters the genomic characteristics of tumors at both the somatic mutation and the copy number variation levels. (iii) CRT-resistant tumors exhibit either a branched or a linear evolution pattern. (iv) Different recurrent mutation signatures in pre-CRT and post-CRT patients implicate mutational processes underlying the evolution of CRT-resistant tumors. (v) High intratumoral heterogeneity in pre- or post-CRT is associated with poor patients' survival.

**Conclusion**: Our study reveals genome landscapes in rectal cancer before and after CRT and tumors evolution under CRT stress. The treatment-associated characteristics are useful for further investigations of CRT resistance in rectal cancer.

## Introduction

Colorectal cancer (CRC) ranks the fifth leading cause of cancer death and approximately 300,000 new CRC cases occur each year in China 1. Rectal cancer accounts for approximately 30% of all CRCs. Concurrent capecitabine, oxaliplatin and radiotherapy followed by total excision of the mesorectum is a mainstay in the management of patients with locally advanced rectal cancer. However, regression of rectal carcinoma following preoperative chemoradiotherapy (CRT) is highly variable 2-5, with the development of relapse and distant metastases being the predominant modes of failure 6, 7. The variety may reflect differences in the biological properties of tumor such as CRT response. Despite the seriousness of this issue, there are few studies on the genome evolution under CRT in locally advanced rectal cancer and the mechanisms of CRT resistance in rectal cancer have not been well characterized. While large genomic studies such as The Cancer Genome Atlas (TCGA) have shed some light on untreated tumors 8, the differences in genome alterations between untreated and treated tumors could be substantial 9-15.

Resistance-related mutations may already exist in tumors before treatment. Or, they may occur and accumulate during the treatment as chemotherapeutic agents and radiation trigger DNA damages in cancer cells 16-19. Thus, profiling the genomic differences and clonal divergence between tumors before and after CRT could reveal effective biomarkers and putative drivers of the resistance to treatment. To this end, we conducted whole-exome profiling of tumor samples collected before and after CRT as well as their matched peripheral blood samples from 28 Chinese individuals with locally advanced rectal cancer. We identified substantial differences in genomic profiles between tumors before and after CRT treatment regarding point mutations (including SNVs short for single nucleotide variants and small INDELs short for insertions and deletions) and copy number variations (CNVs). We found two evolutionary patterns shaping the path from pre-CRT to CRT-resistant rectal cancer and Signature RC Post-2 like mutation signature in post-CRT tumors. Lastly, we found high intratumoral heterogeneity, both before and after CRT, was correlated with shorter survival time in rectal cancer patients.

## Methods

### Study subjects and biospecimen procurement

Patients with histopathologically confirmed locally advanced rectal cancer (*N*=126) were recruited between January 2006 and June 2013 at Cancer Hospital, Chinese Academy of Medical Sciences, Beijing. All patients received preoperative CRT treatment consisting of a total radiation dose of 50 Gy applied in 25 fractions over 5 weeks, concurrent with twice daily administration of capecitabine (825 mg/m^2^ in total) and 50 mg/m^2^/week oxaliplatin. Standardized surgery was performed after an interval of 4 to 6 weeks post CRT. Histological tumor response to CRT was assessed according to the Tumor Regression Grade (TRG) classification 20. Tissue samples with <10% residual tumor cells were defined as good responder (TRG 1 and 2); tissue samples with 10% to 50% residual tumor cells were classified as intermediate responder (TRG 3); and tissue samples with >50% residual tumor cells were considered as poor responder (TRG 4 and 5). Based on these criteria, 65 (51.6%) patients were good responders and the rest 61 (48.4%) were intermediate/poor responders. We then selected those patients with TRG ≥3 and matched triplet samples (i.e., blood, pre-CRT tumor biopsy, and surgically removed post-CRT tumor) for WES. Finally, 28 samples were chosen for analysis after quality control of tissue specimens and DNA samples. Patients' survival time was measured in months from the date of diagnosis to the date of death or last follow-up, respectively. Whether, when and why a patient had died was ascertained through follow-up telephone calls. The last date of follow-up was 25th January 2019 (**Table S1**). Fresh tumor specimens and blood lymphocyte samples were snap-frozen in liquid nitrogen until use. We also selected 22 paraffin-embedded rectal cancer samples from the same 28 patient set for target region sequencing. All patients provided written informed consent before sample collection. This study was approved by the Institutional Review Board of the Chinese Academy of Medical Sciences, Cancer Hospital.

### Whole-exome sequencing

Genomic DNA was extracted from fresh tumor samples and blood samples using the phenol-chloroform protocol, or from formalin-fixed paraffin-embedded (FFPE) tumor samples using QIAamp DNA FFPE Tissue Kit (Qiagen). WES libraries were generated using Agilent SureSelect Human All ExonV5 kit (Agilent) following the manufacturer's instructions. Sequencing was performed by Illunima Hiseq 2500 for paired-end 125 bp reads. In addition, we performed target region sequencing of 78 cancer-associated genes altered in TCGA CRC data set using Agilent SureSelectXT Custom Kits 8, achieving an average coverage of more than 1000X (**Tables S2** and **S3**).

### Analysis of whole-exome sequencing data

DNA reads passing standard quality control were aligned to reference human genome (GRCh37) using Burrows-Wheeler Aligner (BWA) software to get the original mapping results stored in BAM format 21. SAMtools, Picard (http://broadinstitute.github.io/picard/), and GATK were used to sort BAM files and do duplicate marking, local realignment, and base quality recalibration to generate final BAM file 22, 23. ANNOVAR 24 was used to annotate the detected variants, relying on public resources such as dbSNP and 1000 Genome 25, 26. SNVs and small INDELs were detected by VarScan2 27. Exome CNV was used to detect somatic CNV 28. CNVs with coverage ratio >1.3 were considered as gain and <0.7 were considered as loss.

Significantly mutated genes in pre-CRT and post-CRT tumors were inferred by MutSigCV 29 with q<0.2 were considered as driver genes. Broad and focal CNVs were inferred by GISTIC 30. Arms and recurrent focal peaks in amplification or deletion regions were considered significant when q<0.25.

Cancer cell fraction (CCF) of each mutation was calculated by ABSOLUTE integrating mutational allele frequencies and copy number calls 31. Mutations with CCF=1.0 were considered clonal while those with CCF<1.0 subclonal. To identify clonal relationship between pre-CRT and post-CRT tumors, we used PyClone (v0.13.1) to infer the cellular prevalence of mutations based on ABSOLUTE results 32. Clonal phylogeny was visualized using Timescape^33^.

The number of recurrent signatures and the activities of each signature in single samples were inferred by SignatureAnalyzer (v1.1) 34-36 based on all the detected SNVs and the trinucleotide sequence context including the bases immediately 5' and 3' to the mutated base (96 possible mutation types). The inferred mutational signatures were compared with 30 COSMIC signatures using cosine similarity. We calculated the proportion of subclonal mutations in pre- and post-treatment tumor samples cohort separately, and measured it as intra-tumor heterogeneity.

### Statistical analysis

The association between intratumoral heterogeneity and patients' overall/cancer-specific survival was examined using the log-rank test and a multivariate Cox proportional hazards model (including clinical covariates age, gender, KPS, TMN stage, post-chemotherapy and surgical procedure). Both the pre-CRT and the post-CRT tumor sets were divided into two groups based on intratumoral heterogeneity, defined as the proportion of subclonal mutations. The cutoff point was determined using receiver operating characteristic (ROC) curves and the Youden index. Two-sided Wilcoxon tests were conducted to determine whether there were statistically significant differences between two paired abnormal distributions. Two-sided Fisher's exact tests were conducted to check for significant difference of gene mutation frequencies between the TCGA samples and our samples. Spearman rank correlations were calculated between variant allele frequencies (VAFs) of WES and those of the target region sequencing. Correlations were considered significant and positive when *P*<0.05 and r>0.30. A *p*-value of less than 0.05 was used as the criterion for statistical significance. Statistical analyses were performed using R, python or SPSS.

## Results

### Mutations in rectal tumors before and after CRT

All the patients enrolled in this study were treated with the same concurrent CRT followed by total excision of the mesorectum. We selected 28 patients whose post-CRT tumor was classified as resistance to CRT (*N*=28; **Table S1)** and sequenced their pre- and post-CRT tumor samples as well as blood samples. We confirmed each triplet samples belonging to the same individual using NGSCheckMate 37 (**Figure S1** and** Table S4)**. The mean target coverage of WES was 97.6x for tumor samples with 97.3% of the bases >20x and 116.3 for blood samples with 98.4% of the bases >20x (**Table S5**).

The mutation frequencies of our samples were very similar to those of the untreated rectal cancer samples in TCGA (**Table S6**). We identified totally 7,112 somatic mutations from pre-CRT tumor samples and 6,900 somatic mutations from post-CRT tumor samples (**Table S7**). The difference in the number of mutations per sample was not significant (Wilcoxon signed ranks test, *P*=0.716; **Figure S2**). The median coding mutation rates of pre- and post-CRT tumor samples were 1.89 and 1.34 per Mb respectively and the difference was not significant (Wilcoxon signed ranks test, *P*=0.495).

### Recurrent mutations in rectal cancer before and after CRT

**Figure 1:**
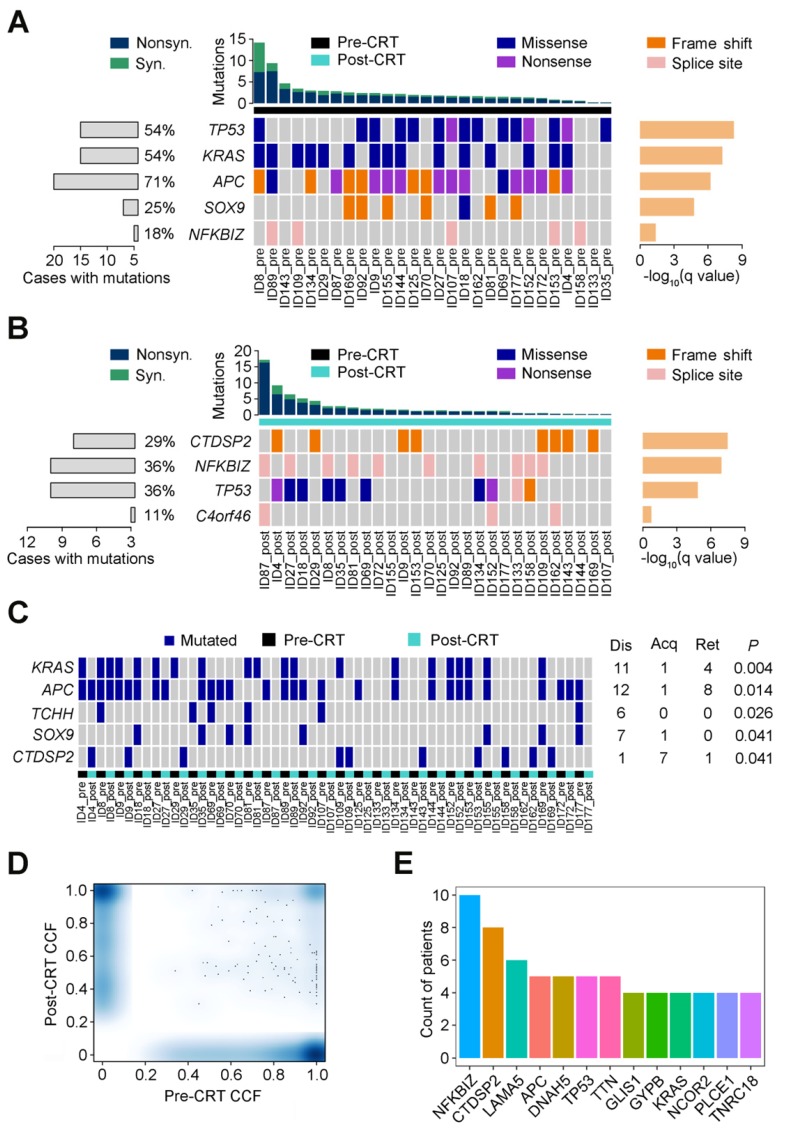
** Recurrent mutations in rectal cancer before and after CRT.** Significantly mutated genes are arranged according to q value (using MutSigCV algorithm). Each column represents a tumor and each row represents a gene. Samples are arranged in the descending order of mutation rate. The bottom center panel demonstrates significant gene landscape. The top panel displays the overall mutation rate in each tumor. The bottom left graph shows percentages of cases with mutations. The bottom right graph denotes -log_10_ (q) for significance level of mutated genes. Samples are divided into pre-CRT set (**A**) and post-CRT set (**B**). Mutation types are shown by colors as indicated. (**C**) Gene mutation counts in post-CRT tumors differ significantly from pre-CRT tumors. Every two columns represent a matched pre- and post-CRT tumor pairs and each row represents a gene. The left heatmap demonstrates gene mutation status (whether or not) in coding regions in 28 pairs of samples. The right graph shows the number of patients with the mutations disappeared (dis), acquired (acq) and retained (ret) after CRT treatment. The *P* value denotes the significance level of the difference in mutation counts between pre-CRT and post-CRT tumors. (**D**) Smoothed color density scatter plot, obtained through a (2D) kernel density estimate, of pre-CRT and post-CRT cancer cell fractions (CCFs) of each mutation in all samples. Dots off the axes indicate mutations in both of pre-CRT and post-CRT tumors while dots on the axes indicate mutations in either pre-CRT or post-CRT tumors. (**E)** Coding mutations in 13 genes were selected under CRT treatment (CCF became higher after CRT) in more than 3 patients.

Using MutSigCV, we identified 5 significant recurrent mutations in pre-CRT tumors, including 4 previously reported colorectal cancer driver genes (*TP53*, *KRAS*,* APC* and *SOX9*) 8 and a novel gene *NFKBIZ* (**Figure 1A**). In contrast, besides *TP53*, *CTDSP2*,* NFKBIZ* and *C4orf46* were significantly mutated in post-CRT tumor samples (**Figure 1B**). In addition, the mutation counts of 5 genes, *KRAS*,* APC*,* TCHH*, *SOX9* and *CTDSP2,* were significantly different in pre- and post-CRT tumors. Three of the 5 genes, *KRAS*,* APC* and *CTDSP2*, had recurrent mutations in more than three patients (**Figure 1C**). Lastly, 13 genes harbored mutations whose CCF was higher in post-CRT tumors than in pre-CRT tumors in more than three patients (**Figure 1D-E**); these mutations may have conferred some selective advantages on cancer cells and made them resistant to CRT treatment. All in all, the three sets of recurrent mutations and their host genes mentioned above might relate to CRT resistance.

### Heterogeneity of mutations and CNVs in tumors before and after CRT

**Figure 2:**
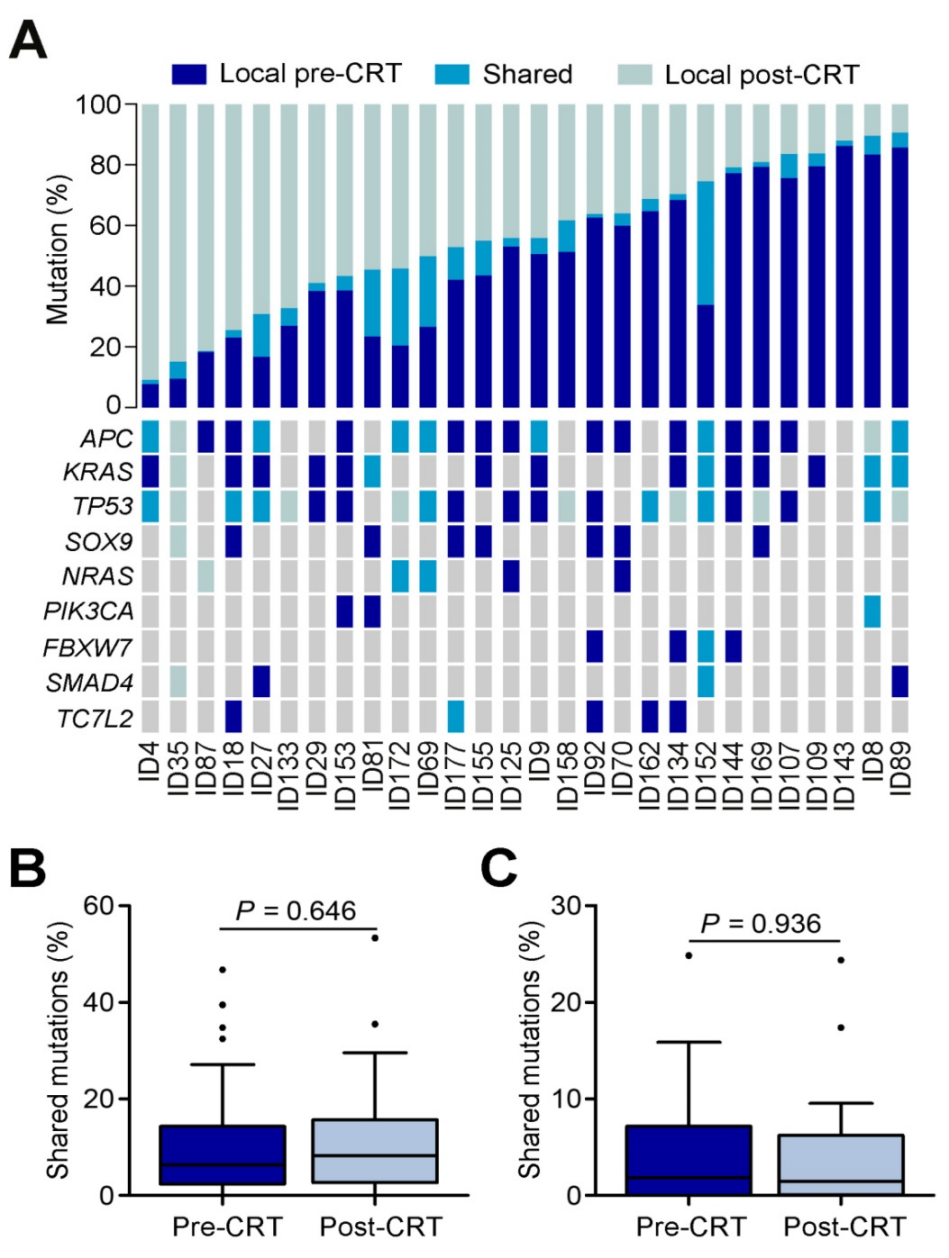
** Mutational heterogeneity in CRT-resistant rectal cancer.** (**A**) Percentages of shared and unique mutations (top) and mutational status (bottom) of selected driver genes reported in TCGA data set of colorectal cancer in matched pre- and post-CRT tumors. Each column represents a matched pre-CRT and post-CRT tumor pairs. (**B**) Boxplot of shared mutations inferred as clonal between pre- and post-CRT tumors. (**C**) Boxplot of shared mutations inferred as subclonal between pre-CRT and post-CRT tumors.

The number of mutations shared by tumor collected before and after CRT ranged from 0.39% to 40.63% with an average of only 8.21% **(Figure 2A)**. A substantial proportion of mutations were unique to tumors before or after CRT, including mutations in previously reported driver genes such as *APC, KRAS, TP53, SOX9, NRAS, PIK3CA, FBXW7, SMAD4 and TC7L2* 8. To make sure these findings were not technical artifacts, we once again extracted DNAs from 22 of the original 28 paraffin-embedded rectal cancer samples (**Table S2**) and then preformed target region sequencing on 78 genes significantly mutated in the TCGA data. The VAFs between target region sequencing and WES were closely correlated (Spearman rank correlation r=0.503,* P*=0.001; **Figure S3** and **Table S3**). The mutations shared by pre- and post-CRT tumors mostly came from the primary clone instead of any sub-clones (**Figures 2B-C**). Furthermore, among the shared mutations, those inferred clonal were enriched in post-CRT tumors while those inferred subclonal were decreased in post-CRT although the differences were not statistically significant (Wilcoxon rank sum test, *P*=0.646 and *P*=0.936, respectively), indicating that tumors under CRT might select mutations beneficial to resistance (**Figures 2B-C**).

**Figure 3:**
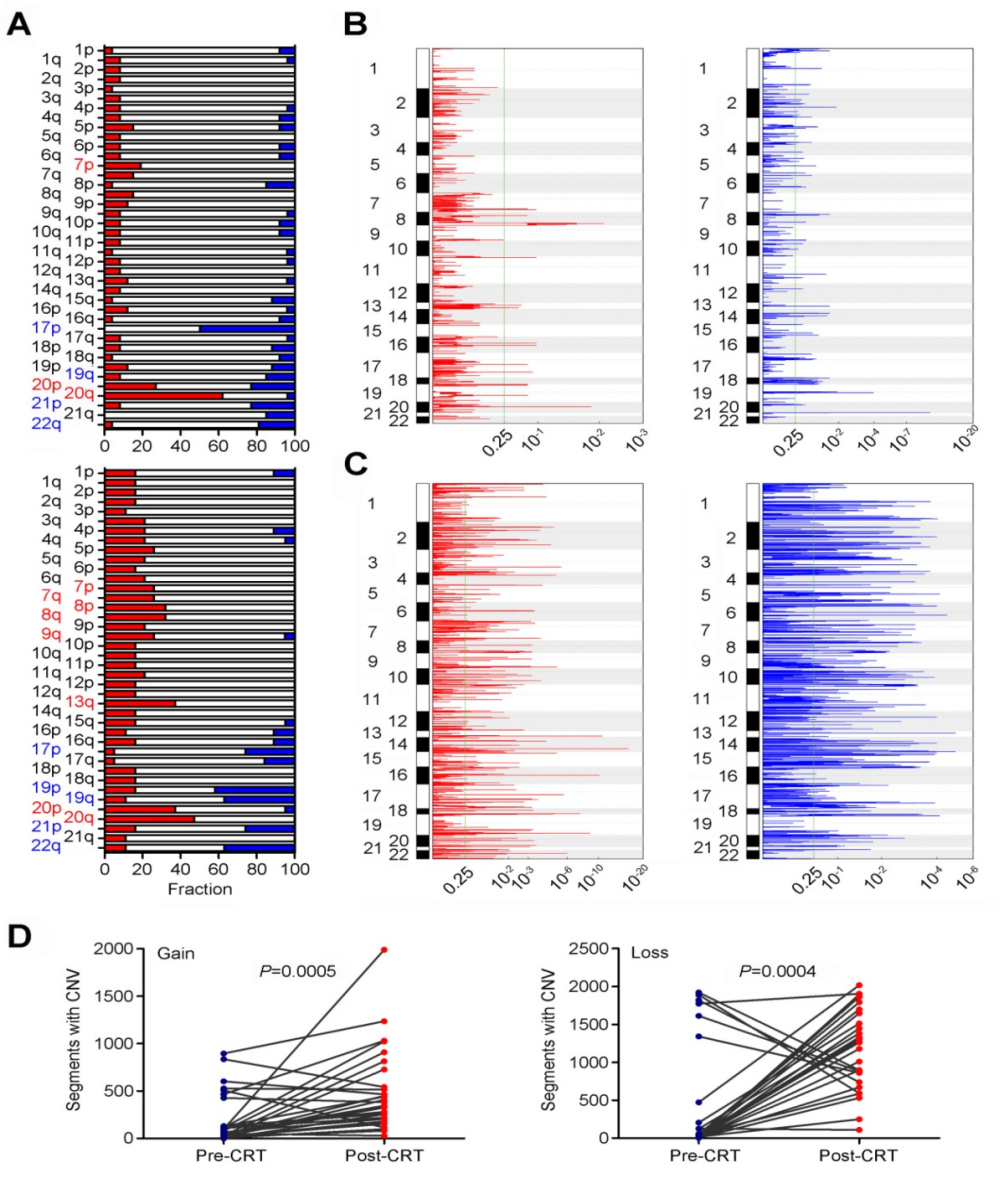
** Comparisons of CNVs in pre- and post-CRT rectal cancer.** (**A**) Broad alterations per chromosome arm. The bar graphs show the frequency of arm-level copy-number alterations in pre-CRT (top) and post-CRT (bottom) tumors. The horizontal axis denotes chromosome arms. The chromosome arms with significant gain or loss (all q<0.25) are shown in red or blue. Focal peaks of amplifications and deletions in (**B**) pre-CRT tumors and (**C**) post-CRT tumors. The x axis represents false discovery rate and the y axis represents chromosome. A dashed line represents the centromere of each chromosome. (**D**) Comparisons of segments with copy number gain and loss between pre-CRT and post-CRT tumors. Segments with copy number gain and loss are significantly higher after CRT treatment (Wilcoxon signed ranks test, *P*=0.0005 and *P*=0.0004, respectively).

We also examined the evolvement of CNVs in rectal cancer under CRT (**Table S8**). In pre-CRT tumors, we identified several arm level changes including loss at 17p, 19q, 20p, 21p and 22q and gain at 7p, 20p and 20q (all q<0.25; **Figure 3A** and **Table S9**). We also identified 14 recurrent amplification peaks and 59 recurrent deletion peaks (both q<0.25; **Figure 3B**). Many genes located in the focal amplification/deletion regions are cancer-associated genes (**Table S10** and **S11**). In post-CRT tumors, we detected loss at 17p, 19p, 19q, 21p and 22q and gain at 7p, 7q, 8p, 8q, 9q, 13q, 20p and 20q (**Figure 3A** and** Table S12**). We found 205 focal amplification regions and 234 focal deletion regions (**Figure 3C** and **Tables S13** and **S14**). Interestingly, the numbers of CNVs in post-CRT tumors were substantially elevated compared with pre-CRT tumors (**Figure 3D** and** Figure S4**). The sharing of CNVs between pre-CRT and post-CRT tumors varied with patients (**Figure S4**). Altogether, these results indicated the heterogeneity between pre- and post-CRT tumors from the same patients characterized by point mutations and CNVs, which may be associated with the resistance to CRT in locally advanced rectal cancer.

### Clonal evolution of rectal cancer under CRT

**Figure 4:**
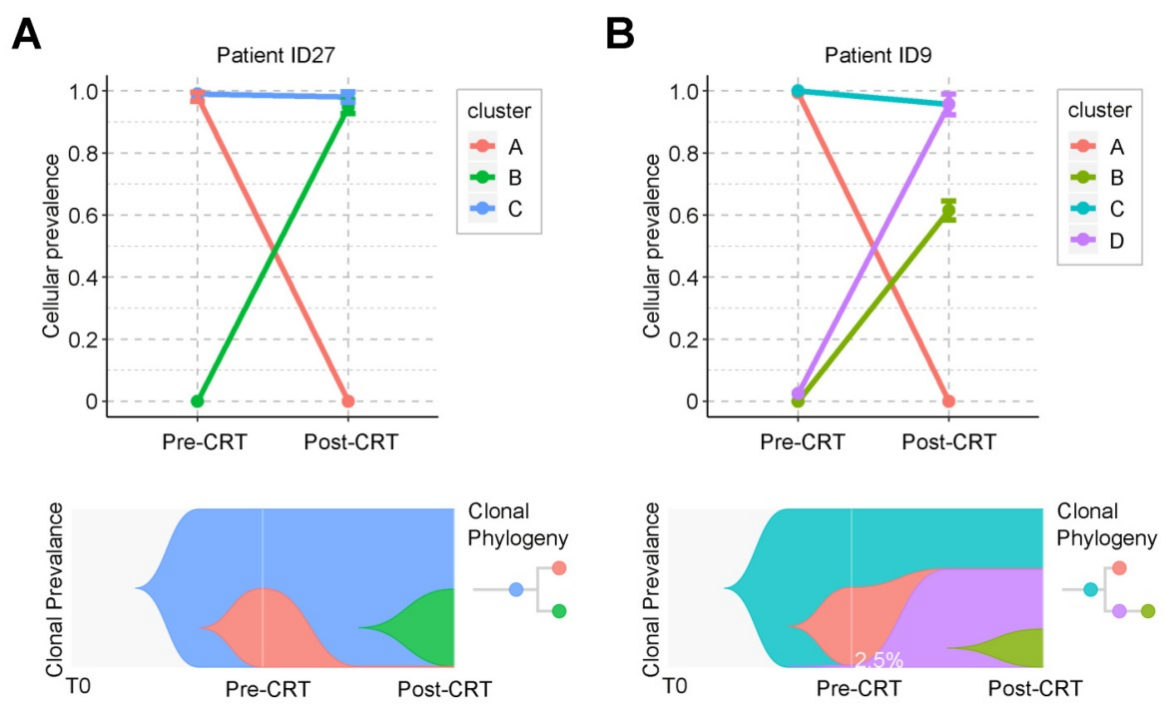
** Graphical representation of clonal evolution from pre- to post-CRT cancer.** (**A**) and (**B**) show two clonal evolution patterns, branched and linear, as observed in two patients, ID27 and ID9, respectively. Top graph shows cellular prevalence of mutations after clustering using PyClone. Bottom graph exhibits the visualization of tumor evolution using Timescape. Each color represents a clone. The horizontal axis denotes 3 different time point of tumor, from the time of tumorigenesis (T0) to diagnosis (pre-CRT) and after surgery (post-CRT). In patient ID9, only 2.5% of cancer cells survived during CRT. See also Supplementary Figure S5.

Clonal analysis identified two evolution patterns of rectal cancer during CRT. One pattern was branched evolution, which we observed in 27 patients. In this evolution mode, the primary clone in the post-CRT tumor did not exist before the treatment. Instead, it was formed during the CRT by some cancer cells that acquired new mutations and developed resistance (**Figure 4A** and **Figure S5**). Take patient ID27 as an example (**Figure 4A**). Three clones were detected during CRT. The ancestor clone (blue) was retained during CRT treatment and possibly associated with carcinogenesis of rectal cancer. One subclone (pink) was almost eradicated after CRT, while the other (green) acquired new mutations during CRT and formed a new clone (green). The tumor mass mainly consisted of the green clones. Another pattern was linear evolution, in which some cancer cells survived under the CRT pressure and grew into the primary clone in the post-CRT tumor. We observed this evolution pattern in only one patient ID9 (**Figure 4B**). Three clones (blue, pink and purple) were detected in the pre-CRT tumor of this patient. During the treatment, the pink clone disappeared, whereas the purple clone, starting with a minor subset of cancer cells (2.5%), became dominant and even developed its own subclone (yellow-green).

### Mutations driving the evolution of resistant rectal cancer

**Figure 5:**
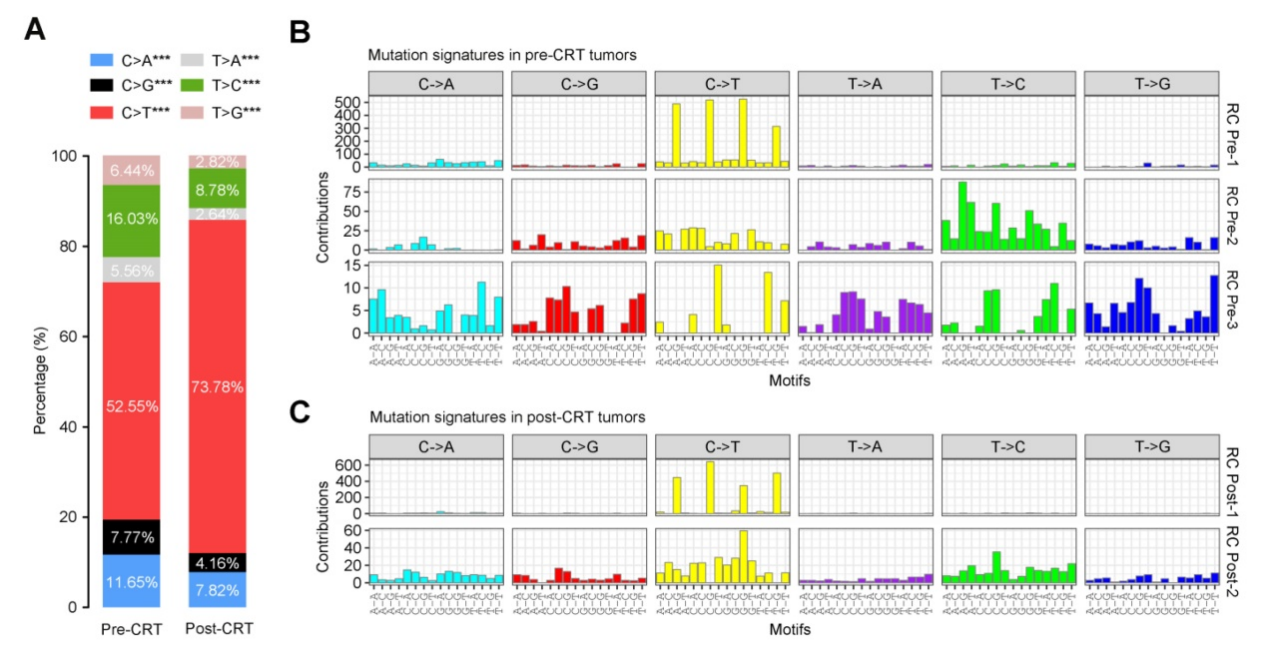
** Mutational signatures in pre- or post-CRT rectal cancer.** (**A**) Stacked bar graph displays the percentages of 6 possible substitutions for single nucleotide variations in pre-CRT (left) and post-CRT (right) tumors. ***, *P*<0.001. Three and two de novo signatures are identified in (**B**) pre-CRT and (**C**) post-CRT tumors, respectively. Signature RC Post-2 may be associated with CRT resistance.

To elucidate how the mutational signatures evolve in rectal cancer treated with concurrent CRT, we then analyzed the distribution of six types of SNVs (C>A, C>G, C>T, T>A, T>C and T>G) and observed significantly more increase in C>T transition in post-CRT tumors than in pre-CRT tumors (**Figure 5A**). Further characterization of the mutational effect of CRT using a Bayesian variant of the non-negative matrix factorization (NMF) algorithm identified 3 signatures in pre-CRT tumors and 2 signatures in post-CRT tumors (**Figure 5B-C**), 4 of which closely resemble COSMIC signatures 34-36, 38-40 as shown in **Figure S6**. Signature RC Pre-1 in pre-CRT tumors was characterized by C>T transition and similar to COSMIC Signature 1 for age-related accumulation of 5-methylcytosine deamination events. Signature RC Pre-2 seemed to be a combination of several COSMIC signatures including two found in liver cancer. Signature RC Pre-3 did not resemble any COSMIC signatures and appeared to be a novel signature in rectal cancer (**Figure S7A**). In post-CRT tumors, Signature RC Post-1 also resembled COSMIC Signature 1 while Signature RC Post-2 was similar to COSMIC Signature 5, characterized by a broad spectrum of base changes and present in all tumor types. Analysis of signature activities showed a fair number of mutations attributable to Signature RC Post-2 (**Figure S7B**), indicating that this mutational signature could be associated with the resistance to CRT and shape the evolution of resistant rectal cancer.

### Intratumoral heterogeneity is correlated with survival time in patients

**Figure 6:**
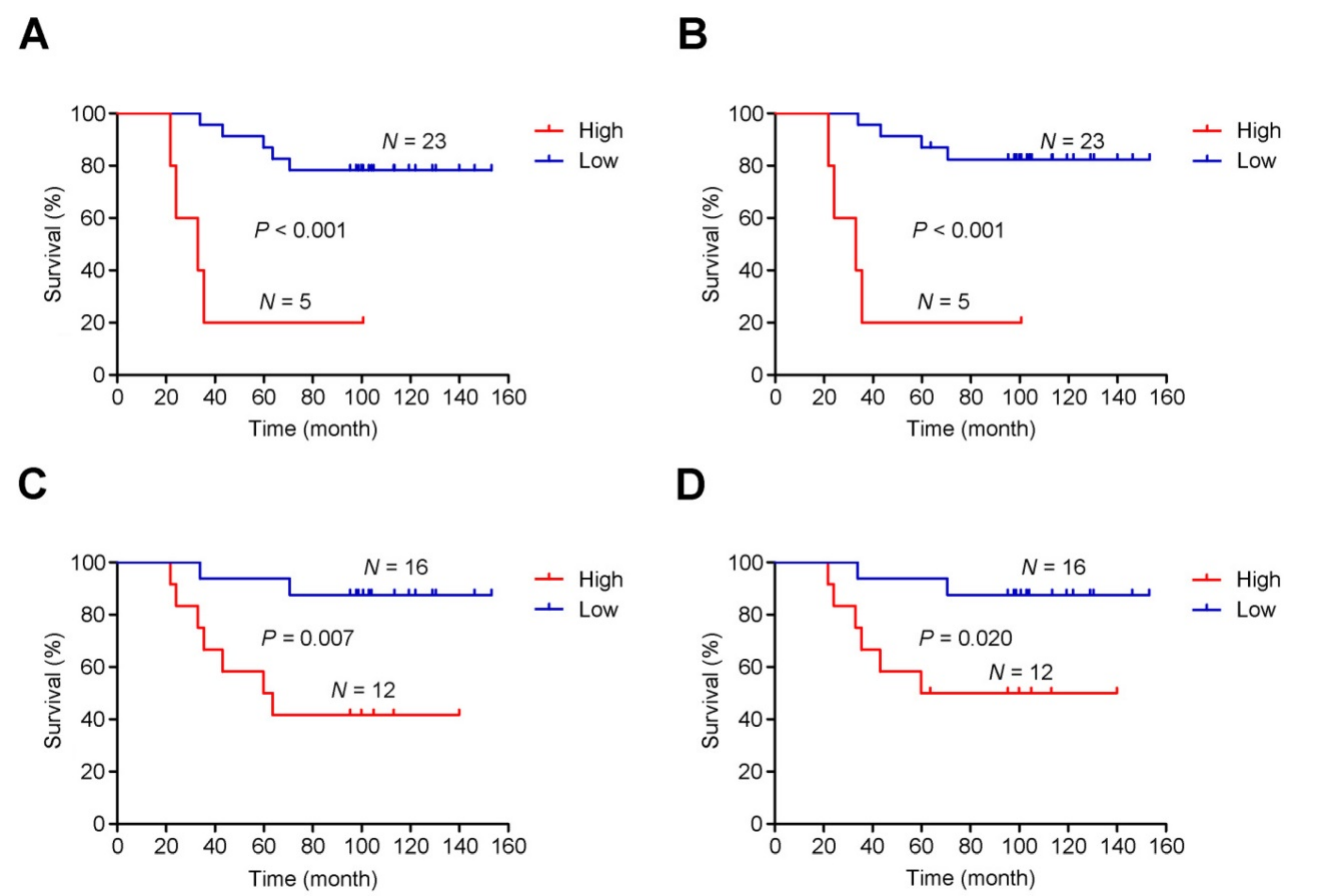
** Association between intratumoral heterogeneity and survival.** The cumulative risk of intratumoral heterogeneity is shown by the Kaplan-Meier survival curves between patients with low intratumoral heterogeneity (blue) and patients with high intratumoral heterogeneity (red): (**A**) Overall survival in pre-CRT patients, (**B**) Cancer-specific survival in pre-CRT patients, (**C**) Overall survival in post-CRT patients, and (**D**) Cancer-specific survival in post-CRT patients.

As mentioned, post-CRT tumors developed genome-wide differences from pre-CRT tumors, including extensive CNVs. Thus, instead of focusing on specific gene or region targets, we examined the association between intratumoral heterogeneity (defined as the proportion of subclonal mutations in a tumor sample) 41 and patients' survival time. We found a shorter survival time was significantly associated with higher intratumoral heterogeneity of either pre-CRT (*P*_log-rank_<0.001 for both overall and cancer-specific survival; **Figure 6A-B**) or post-CRT tumors (*P*_log-rank_=0.007 for overall survival and *P*_log-rank_=0.020 and cancer-specific survival; **Figure 6C-D**). Cox proportional hazards model analyses showed that after adjusting for potentially confounding factors such as age, sex, KPS, and TNM, an excess hazard ratio (HR) for death was still associated with the heterogeneity levels in either pre-CRT tumors (HR=35.44, 95% CI=3.39-370.74, *P*=0.003 for overall survival; HR=57.28, 95% CI=4.06-808.63, *P*=0.003 for cancer-specific survival) or post-CRT tumors (HR=6.90, 95% CI=1.28-37.26, *P*=0.025 for overall survival; HR=5.24, 95% CI=0.93-29.40, *P*=0.060 for cancer-specific survival).

## Discussion

In this study, we conducted a WES profiling of locally advanced rectal cancers that develop resistance to preoperative CRT. Matched samples collected from the same individuals in the same ethnic group largely reduced genetic noise. Longitudinal study design (the same tumor sample collected before and after CRT) allows us to identify mutational characteristics and evolutionary patterns likely associated with CRT resistance.

We obtained several interesting results in this study. Firstly, we not only confirmed reported mutations in *TP53*, *APC*, *SOX9* and* KRAS*, but also identified a novel significantly mutated gene, *NFKBIZ,* in rectal cancer. *NFKBIZ* encodes IκBζ, an atypical member of the nuclear IκB family of proteins, which is an important regulator of inflammation, cell proliferation and survival 42-44. In addition, mutations in *CTDSP2*, *APC*, *KRAS*, *TP53* and *NFKBIZ* were suggestively associated with CRT resistance because *KRAS* and *TP53* are known colorectal cancer driver genes and key players in preoperative CRT resistance 45-50. However, the role of *CTDSP2* has so far remained unclear. As a FOXO target gene it encodes C-terminal domain small phosphatase, which has been reported to regulate cell cycle progression through RAS and p21Cip1/Waf1 51. All of the acquired and retained mutations in *CTDSP2* were frame shift deletions, occurring at the 213 amino acid position. *CTDSP1/2* are the host genes of miRNA-26a/b, which can inhibit the phosphorylated form of RB 52 and hypo-phosphorylation of RB is known to cause drug resistance by activating the mTOR-AKT pathway 53. Given that *CTDSP2* is rarely mutated in the untreated TCGA rectal cancer set (2/132), its mutation, along with *APC, KRAS*, *TP53* and *NFKBIZ* mutations, might help predict CRT resistance in locally advanced rectal cancer.

Secondly, we revealed genomic differences between tumors before and after CRT treatment from the same individuals regarding point mutations and CNVs. The majority of somatic mutations identified in our patient cohort were exclusive to tumors either before or after CRT treatment: only 8.21% of mutations on average were shared by both. Clonal analysis revealed that the shared clonal mutations increased while shared subclonal mutations decreased after CRT. Pre- and post-CRT tumors also showed very different CNVs. We found that high intratumoral heterogeneity was associated with poor survival time in patients, likely because high intratumoral heterogeneity endows tumors with the genetic variation fueling tumor clone evolution under the selection pressure from CRT 54. These findings provide additional information for prognostic prediction of CRT resistance.

Thirdly, we identified several recurrent mutational signatures in pre- and post-CRT tumor samples. Among them, Signature RC Post-2 is suggestively associated with resistance to CRT. We also identified two evolutionary patterns in CRT-resistant tumors.

Due to the difficulty in collecting matched triple samples (pre-CRT tumor, post-CRT tumor and blood) from the same individual, the sample size of this study is limited. A larger sample size would increase the detection power. Moreover, it would be interesting and needed to compare our results with those from studies conducted in other ethnic groups to pinpoint population-specific therapeutic biomarkers for CRT. In addition, whole-genome sequencing, which by theory would disclose more genomic variants (e.g., non-coding mutations and structural variations) relevant to CRT resistance, is warranted in future studies. In summary, we believe that the present study has laid a solid stepping stone to future investigations.

## Abbreviations

CCF: cancer cell fraction
COSMIC: catalogue of somatic mutation in cancer
CNVs: copy number variations
CRC: colorectal cancer
CRT: chemoradiotherapy
FFPE: formalin-fixed paraffin-embedded
KPS: Karnofsky performance score
NMF: non-negative matrix factorization
ROC: receiver operating characteristic
SNVs: single nucleotide variants
TCGA: the cancer genome atlas
TRG: tumor regression grade
VAFs: variant allele frequencies
WES: whole-exome sequencing
