# Supplementary material for: Genome landscapes of rectal cancer before and after preoperative chemoradiotherapy
Source: Theranostics. 2019 Sep 21;9(23):6856–66. doi: 10.7150/thno.37794 (PMC6815957; doi:10.7150/thno.37794)
Supplement: Supplementary file 1 — Supplementary figures. [file thnov09p6856s1.pdf]

Supplementary Figure

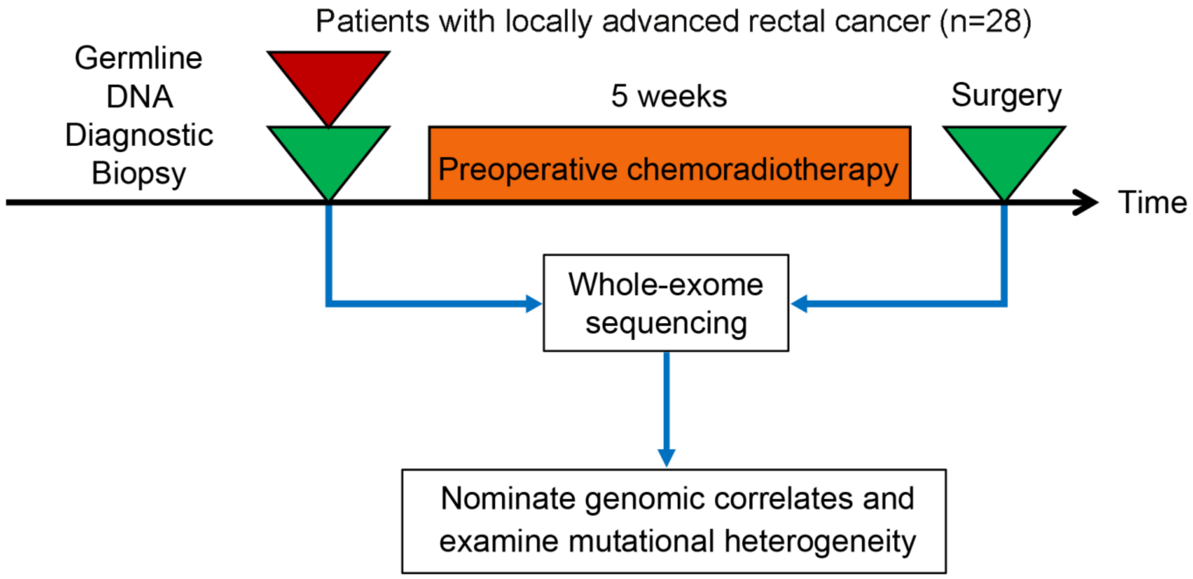

Supplementary Figure S1. Workflow of study design.

Tumor samples before and after chemoradiotherapy (CRT) and blood samples before CRT were collected from 28 patients, who received standard CRT (see Methods). Based on the Tumor Regression Grade (TRG) classification, all these patients were classified as nonresponders to CRT. Whole-exome sequencing (WES) and genome alteration analysis were performed on these samples.

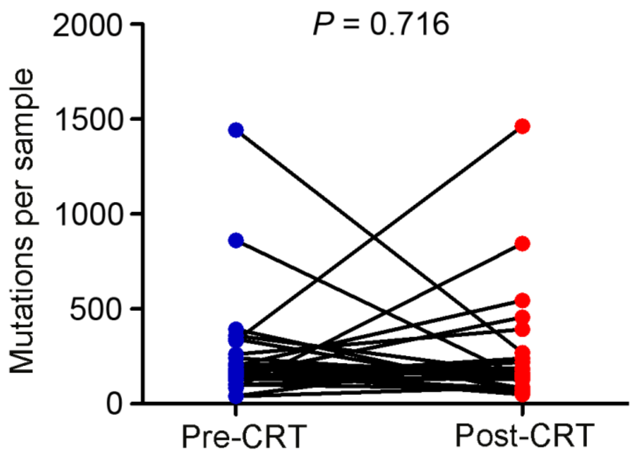

Supplementary Figure S2. Comparison of mutation burden in tumors before and after CRT.

The number of mutationsper sample in pre-CRT and post-CRT tumors are not statistically different, with *P* = 0.716 by Wilcoxon signed ranks test.

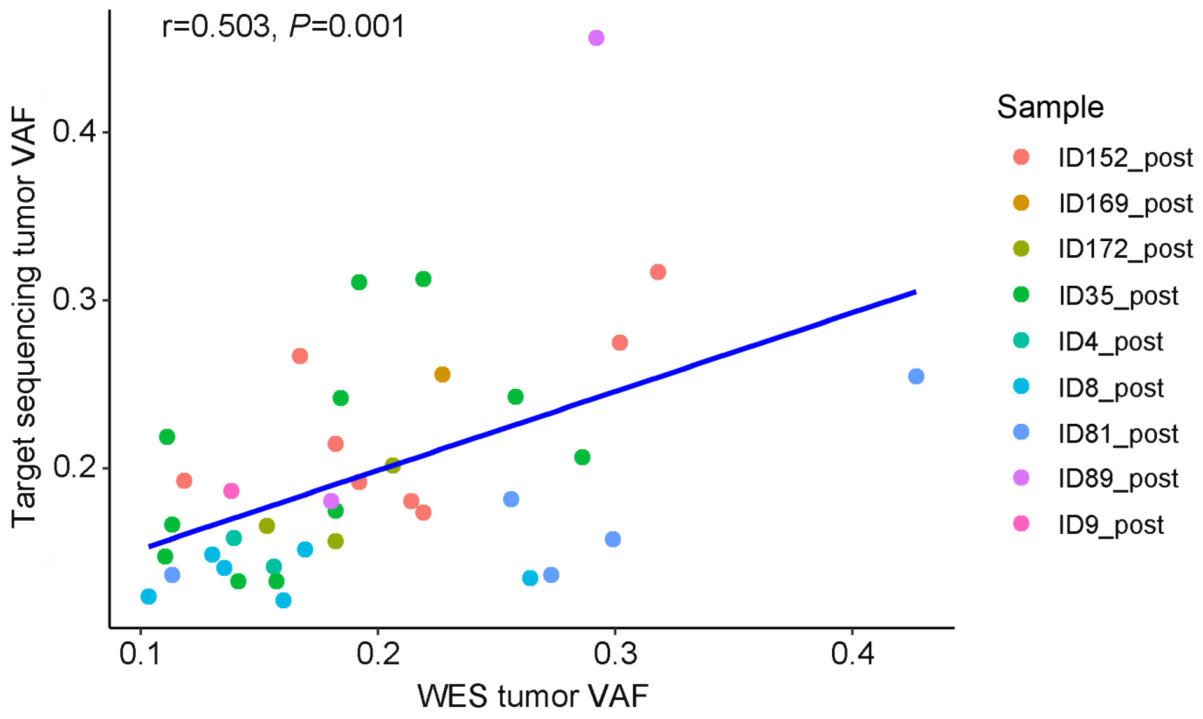

Supplementary Figure S3. Correlation of VAFs between WES and target region sequencing.

Each dot represents a somatic mutation and each color corresponds to a paraffin-embedded sample. Values are variant allele frequencies (VAF) resulted from WES or target region sequencing. The VAF is defined as the ratio between the number of reads supporting the alternative base and the total number of reads coverings.

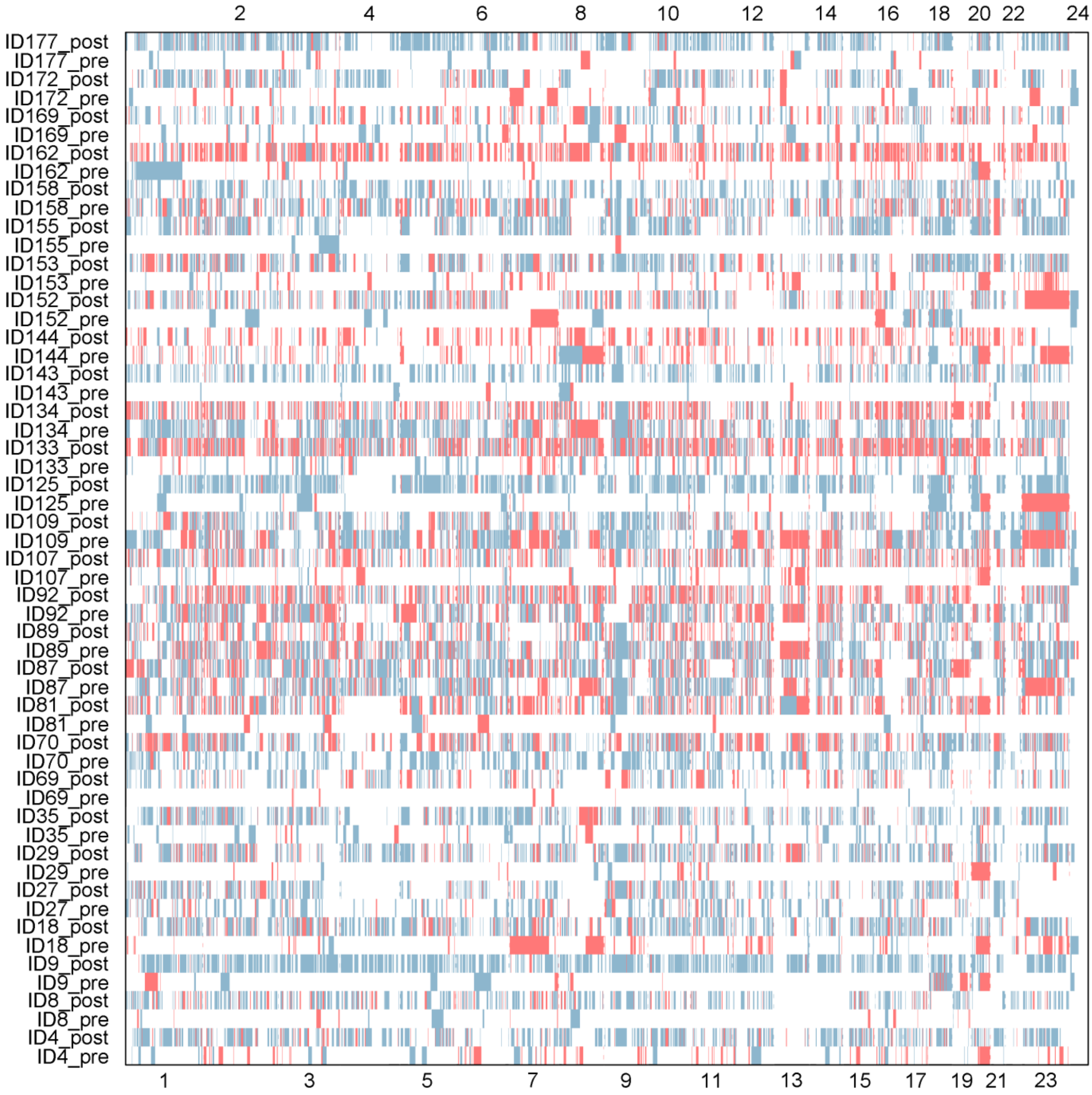

Supplementary Figure S4. CNV profiles in pre-CRT and post-CRT tumors

Matched pre-CRT and post-CRT tumors are arranged together and every two rows represent a pair of pre-CRT and post-CRT tumors. Each column represents chromatin position. Copy number gains are showed in red while losses are showed in blue.

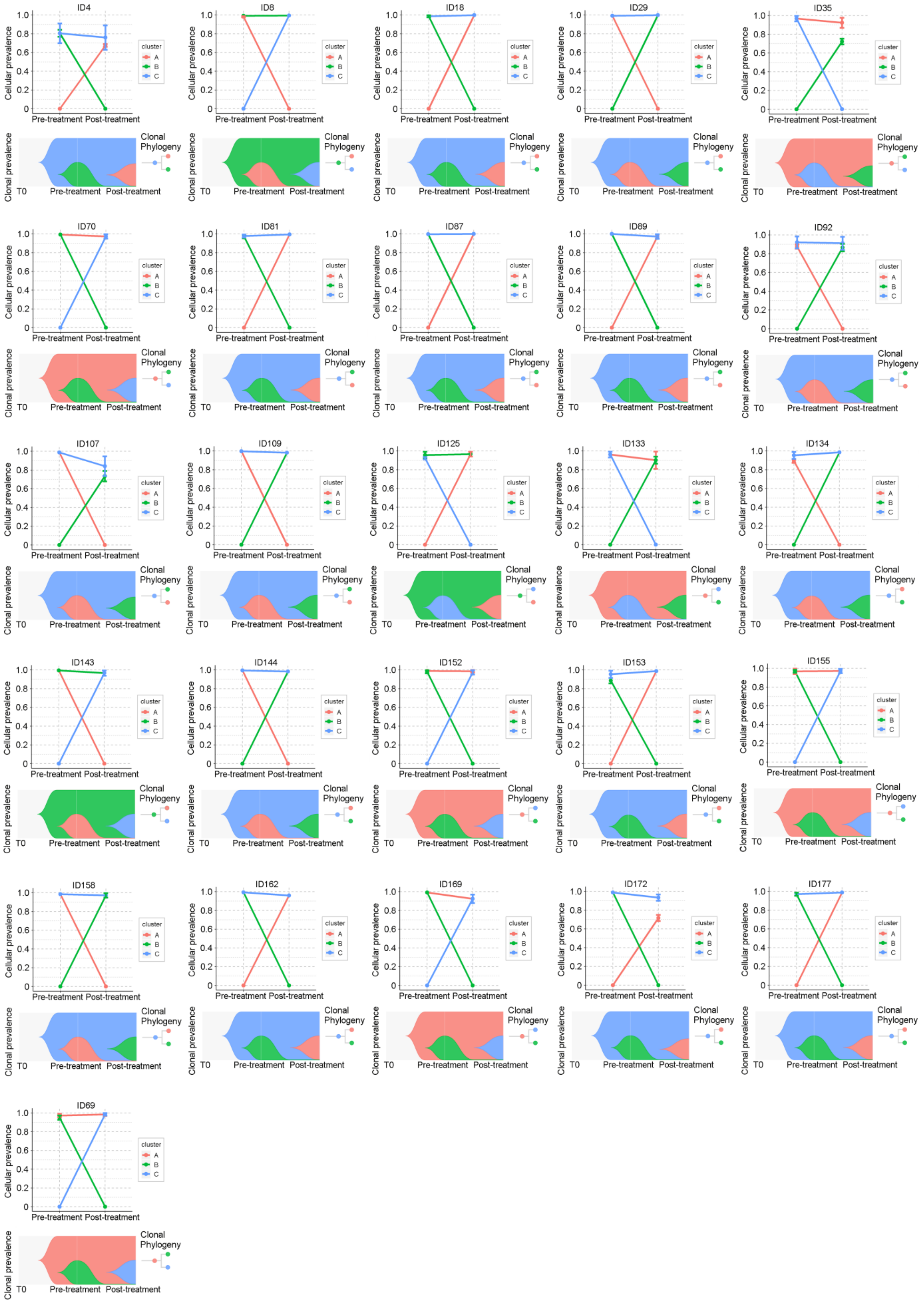

Supplementary Figure S5. Graphical representation of clonal evolution of 26 patients.

This figure is related to Figure 4 in the text. Cellular prevalence of mutations after clustering and clonal phylogeny in matched pre-CRT and post-CRT tumors from another 26 patients, all of them belong to branched evolution pattern.

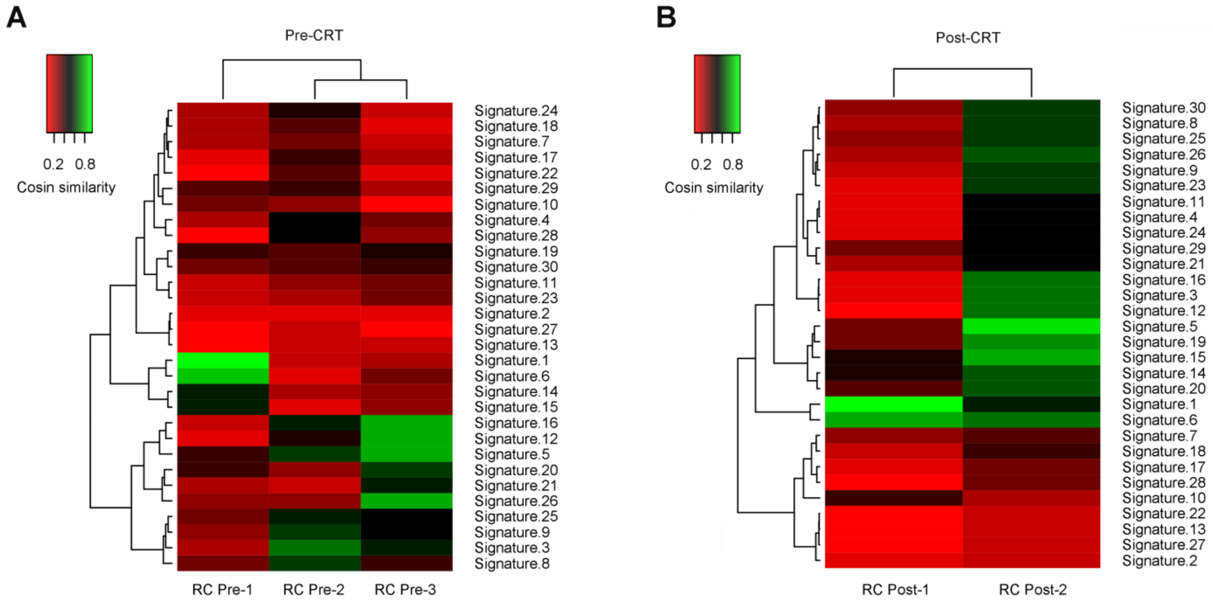

Supplementary Figure S6. Cosine similarity of signatures in COSMIC and our rectal cancer.

Unsupervised hierarchical clustering of signatures identified in (**A**) pre-CRT and (**B**) post-CRT tumor sets and the 30 signatures described by COSMIC based on cosine similarity.

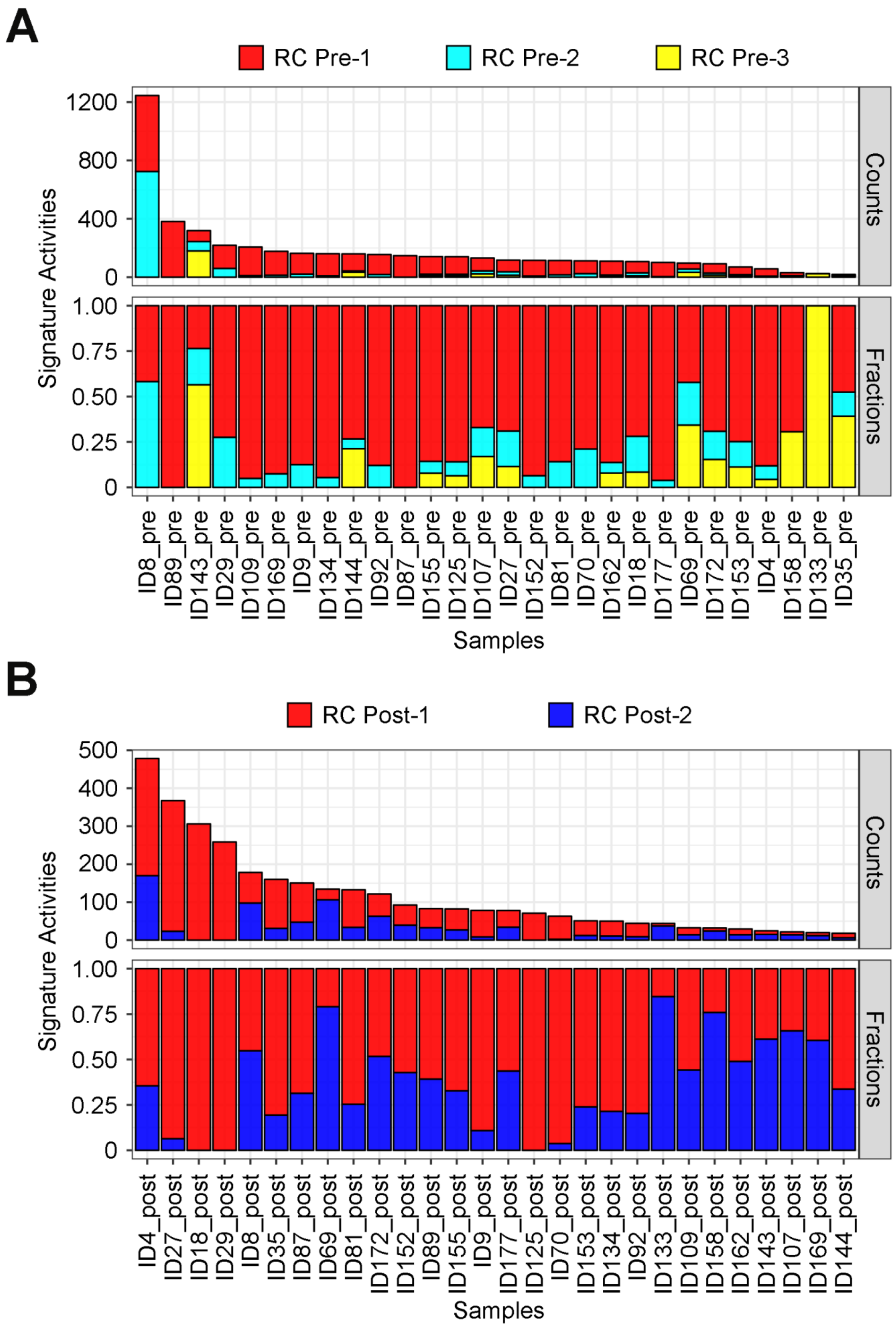

Supplementary Figure S7. The activity of the identified signatures across samples

Overall mutation counts and fractions of signature associated mutations for (**A**) pre-CRT and (**B**) post-CRT tumors.
